# Dual Immunotherapy for Pericardial Mesothelioma That Developed After a Decade‐Long Idiopathic Pericarditis: A Case Report

**DOI:** 10.1002/rcr2.70190

**Published:** 2025-05-26

**Authors:** Beatriz Grau Mirete, Paula Rodriguez Paya, Mariano Martinez Marin, Asia Ferrández Arias, Miguel Borregón Rivilla, Antonio David Lazaro Sanchez, Pedro Morillas Blasco, Alvaro Rodriguez‐Lescure, Javier David Benítez Fuentes

**Affiliations:** ^1^ Department of Medical Oncology Hospital General Universitario de Elche, FISABIO Elche Spain; ^2^ Department of Medical Oncology Hospital General Universitario Morales Meseguer Murcia Spain; ^3^ Department of Cardiology Hospital General Universitario de Elche Elche Spain

**Keywords:** case report, dual immunotherapy, idiopathic pericarditis, immune checkpoint, pericardial mesothelioma

## Abstract

Malignant pericardial mesothelioma (MPeM) is exceptionally rare, and its association with long‐standing idiopathic pericarditis remains unreported. We present a 46‐year‐old woman with a decade‐long history of recurrent idiopathic pericarditis who developed multiple pericardial nodules, mediastinal lymphadenopathy, and metastatic liver and bone lesions. A core needle biopsy confirmed biphasic MPeM. Due to her pericardial condition, she initially received platinum‐based chemotherapy instead of immunotherapy, but the disease progressed. She then underwent dual immune checkpoint inhibition (ICI) and achieved a significant clinical and radiological response. This case raises the possibility of chronic inflammation contributing to pericardial malignancy and underscores the potential role of dual ICI in this rare and challenging disease.

## Graphical abstract

We describe a patient with recurrent idiopathic pericarditis who, after more than a decade, developed a biphasic metastatic malignant pericardial mesothelioma and responded to dual immune checkpoint inhibition.

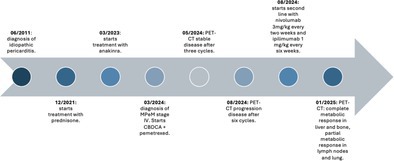

## 1 Introduction

Malignant pericardial mesothelioma (MPeM) is an exceptionally rare and aggressive malignancy, representing less than 1% of all mesotheliomas [1]. It often presents with pericardial effusion or nonspecific cardiac symptoms, leading to misdiagnosis and late‐stage detection, often postmortem. Diagnosis is established by histopathological examination, and it is classified into three subtypes: epithelioid, sarcomatoid, and biphasic. Prognosis remains poor, with a median survival of 6 months [1]. Here, we describe a patient with recurrent idiopathic pericarditis who, after more than a decade, developed a biphasic metastatic MPeM and responded to dual immune checkpoint inhibition (ICI).

## 2 Case Report

**FIGURE 1:**
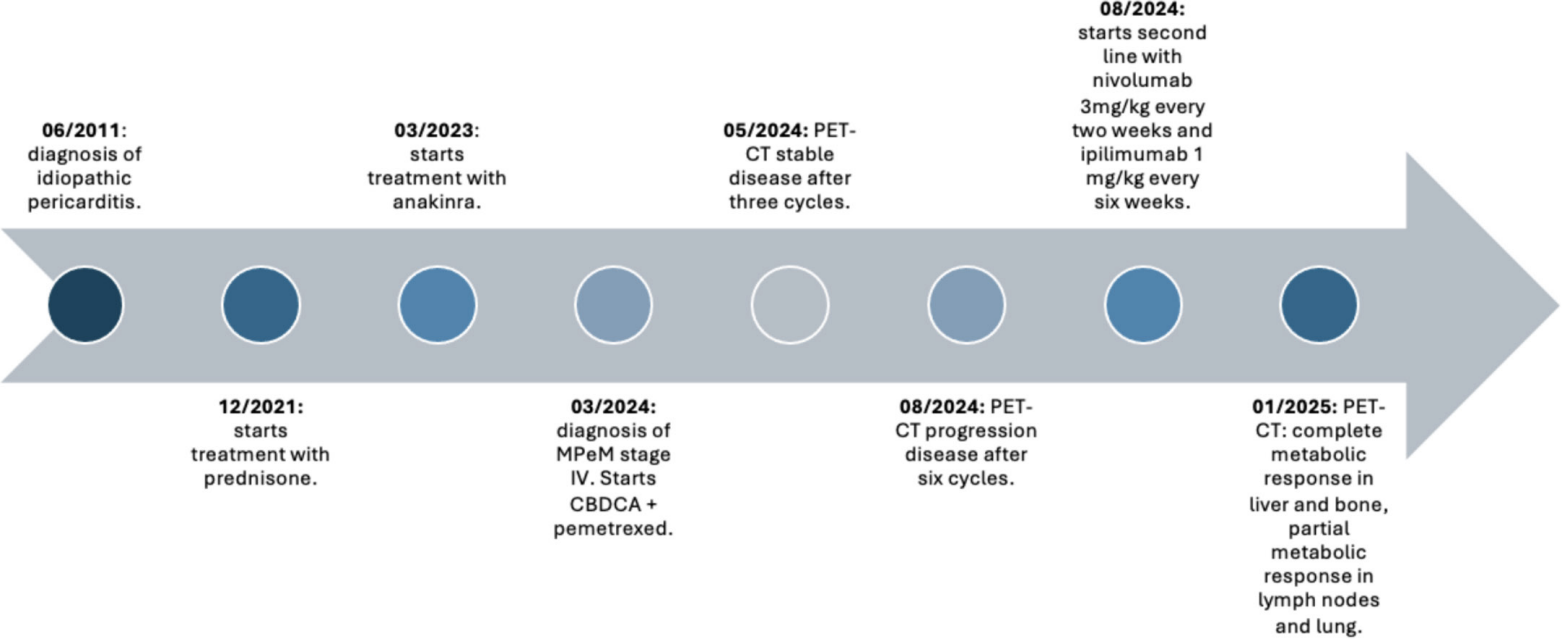
Timeline of events.

The patient, a 46‐year‐old obese Caucasian woman with no asbestos or tobacco exposure, developed recurrent idiopathic pericarditis in 2011, presenting with fever and chest pain. Diagnostic evaluation at that time revealed a severe pericardial effusion and hypermetabolic pericardial thickening on PET‐CT imaging. She initially improved with nonsteroidal anti‐inflammatory drugs (NSAIDs), but frequent flare‐ups led to prednisone (10 mg/day) in December 2021. After two failed tapering attempts, anakinra (100 mg/day) was added in March 2023, improving her condition. A timeline of the patient's clinical events is illustrated in Figure 1.

In March 2024, a routine follow‐up PET‐CT revealed new pericardial nodules, mediastinal adenopathy, bilateral pulmonary nodules, and metastatic lesions in the liver (segment IVa) and L5 vertebra. A liver core needle biopsy confirmed biphasic MPeM with expression of programmed cell death protein ligand (PD‐L1) (sp263) of 30%, positive for CKAEI/AEIII and calretinin, but negative for WT1, S‐100, CD45, P40, CK7, CK20, CDX2, and TTF1. Next‐generation sequencing (NGS) via Oncomine Comprehensive Assay v3 identified a MYCN (p.Ala231del) deletion of unknown significance.

**FIGURE 2:**
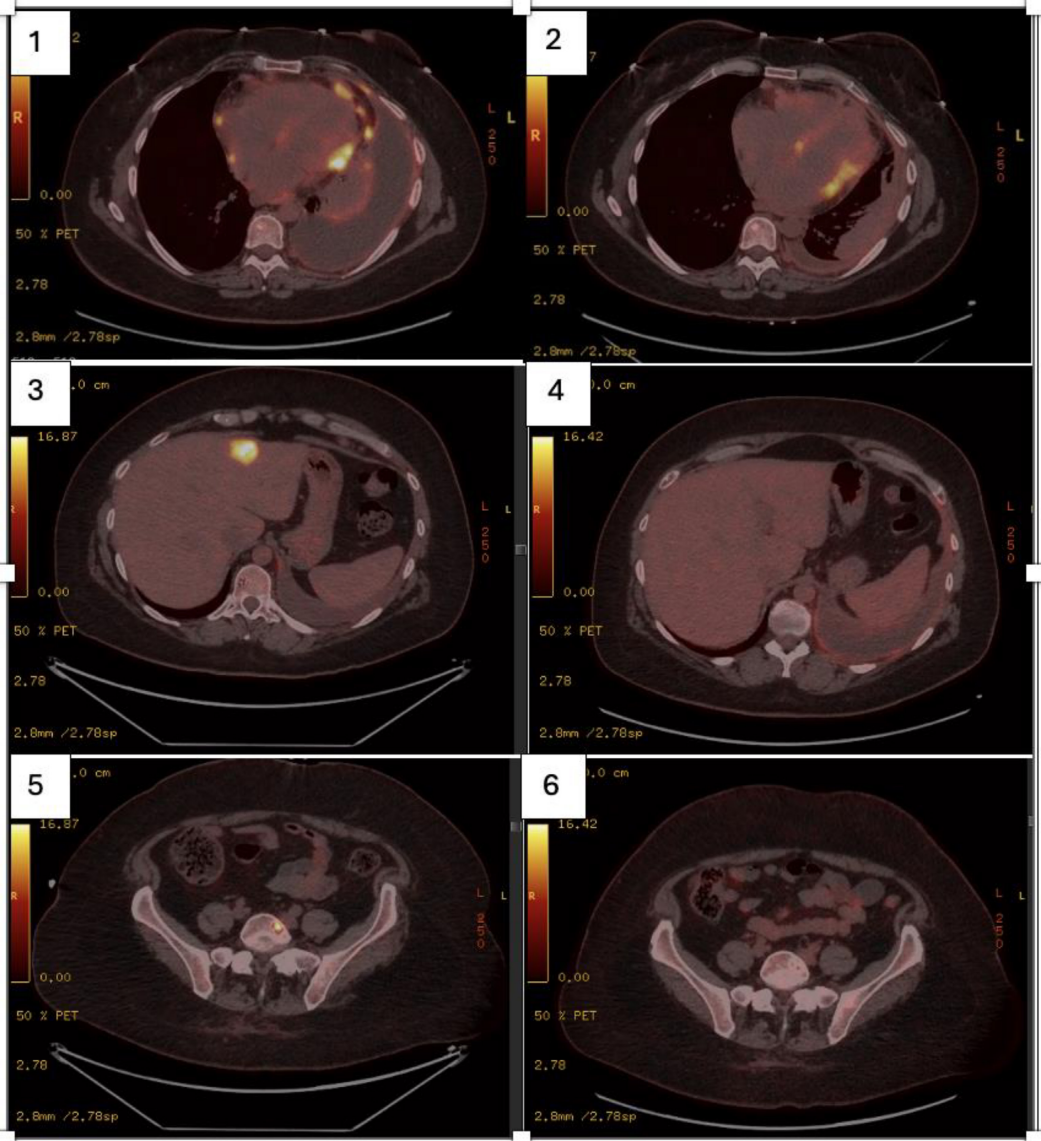
Metabolic response to dual immunotherapy in malignant pericardial mesothelioma. PET‐CT scans before (1, 3, 5) and after (2, 4, 6) three cycles of nivolumab and ipilimumab. Panels 1 and 2 show pericardial nodules and pleural effusion. Panels 3 and 4 depict liver lesions, demonstrating a complete metabolic response post‐treatment. Panels 5 and 6 illustrate bone lesions, also showing a complete metabolic response.

Anakinra was discontinued after diagnosis. Given concerns that immunotherapy might worsen idiopathic pericarditis, first‐line treatment began with carboplatin (AUC 4) and pemetrexed (500 mg/m^2^) every 21 days, supplemented with folic acid and vitamin B12. After three cycles, the disease stabilised, but progression occurred after six, with new bone and liver lesions. In August 2024, due to poor prognosis and limited treatment options, a risk–benefit evaluation supported second‐line dual ICI despite the underlying pericarditis. The patient agreed to nivolumab (3 mg/kg every 2 weeks) and ipilimumab (1 mg/kg every 6 weeks). The first cycle was well tolerated under close monitoring. After three cycles, PET‐CT showed a complete metabolic response in liver and bone lesions and a partial response in most mediastinal and pulmonary nodules. Pericardial response was unassessable due to pre‐existing hypermetabolic nodules (Figure 2).

## 3 Discussion

The role of asbestos exposure in the pathogenesis of MPeM remains controversial, with no definitive correlation established [1]. While evidence directly linking reactive proliferation or recurrent pericarditis to MPeM is lacking, chronic inflammation, through dysregulated proinflammatory mediators, cytokines, and oxidative stress, could plausibly contribute to malignant transformation [2]. In this patient, a decade‐long history of recurrent idiopathic pericarditis and treatment‐related immunosuppression were likely predisposing factors. However, it is not possible to determine exactly when malignant transformation began. The first indication of MPeM was detected via PET‐CT, which showed new pericardial nodules, mediastinal lymphadenopathy, bilateral pulmonary nodules, and metastatic lesions in the liver. Given the aggressive nature of this malignancy, it is reasonable to assume that the malignancy had not been present for an extended period before these findings.

Due to the rarity of MPeM, there is a lack of prospective studies and specific clinical guidelines for its management. Current treatment recommendations are extrapolated from data of patients with malignant pleural mesothelioma (MPM), although the efficacy of these approaches in MPeM remains uncertain. Platinum‐based chemotherapy offers limited survival benefits, typically extending life by only a few months [1]. The CheckMate 743 trial [3], which excluded MPeM, demonstrated superior survival outcomes with nivolumab and ipilimumab over chemotherapy in MPM. Although it remains uncertain whether PD‐L1 status or histologic subtype behaves as a primary driver of ICI response, our patient with biphasic MPeM and PD‐L1 at 30% showed a favourable response to dual ICI [3]. Despite direct evidence for dual ICI in MPeM is lacking, the favourable clinical outcome in this case suggests it may be a viable option, particularly for patients with PD‐L1‐positive, non‐epithelioid histologies. However, given the rarity of MPeM, only isolated case reports have documented outcomes of treating these diseases with ICIs (Table 1).

**TABLE 1:** Summary of published case reports on immune checkpoint inhibitors in malignant pericardial mesothelioma.

Study	Patient (age/sex, exposure)	Histology	Immunotherapy regimen (line of therapy)	Objective response	Survival outcome
Arponen et al. [4]	Mid‐20s female; no asbestos exposure	Epithelioid	Pembrolizumab 200 mg Q3W (second‐line); atezoliazumab 1200 mg/m^2^ (third‐line)	Stable disease	Survival ~4.5 years since first‐line treatment with platinum‐based chemotherapy
Fujiwara et al. [5]	36‐Year‐old male; no asbestos exposure	Biphasic	Nivolumab (first‐line)	Not reported	The patient died 48 days since first dose of nivolumab (of a total of 2 doses)
Gong et al. [6]	62‐Year‐old female; no asbestos history	Epithelioid	Pembrolizumab 200 mg Q3W + carboplatin/pemetrexed (first‐line)	Partial response	Alive after 5 months of ICI initiation
Seal and Simon [7]	74‐Year‐old male; history of asbestos exposure	Epithelioid	Nivolumab and ipilimumab (first‐line)	Not reported	Not reported

Notably, one young female patient treated with pembrolizumab (second‐line, with subsequent atezolizumab) attained stable disease and long‐term survival (> 4.5 years) [4], whereas a 36‐year‐old male with biphasic disease treated with first‐line nivolumab experienced rapid progression [5]. Additionally, a case combining pembrolizumab with chemotherapy in a 62‐year‐old female resulted in a partial response with disease control at 5 months [6]. A report of first‐line dual ICI in a 74‐year‐old male also supports the potential for immunotherapy, however detailed outcomes were not provided [7]. Collectively, these cases suggest that ICIs may offer a survival benefit in select patients with MPeM.

Due to the rarity of mesothelioma, its genomic landscape remains poorly studied. While NGS has identified potentially actionable alterations, the effectiveness of targeted therapies remains limited. Mesothelioma is primarily characterised by inactivating mutations in tumour suppressor genes such as BAP1, CDKN2A, NF2, SETD2, and TP53, while activating oncogene mutations are rare [8]. In this case, a deletion was identified in the MYCN oncogene; however, there is insufficient evidence to determine its pathogenicity. Approximately 1%–2% of patients have molecular alterations potentially targetable with therapies like ALK rearrangements, which hold clinical relevance. Poly ADP‐ribose polymerase inhibitors (PARPi), including olaparib and rucaparib, have shown mixed results in BAP1‐mutated cases, while CDK4/6 inhibitors, such as abemaciclib, have been explored for CDKN2A alterations [8].

Patients with a history of autoimmune diseases (AD) were excluded from CheckMate 743, where 30% of participants in the immunotherapy arm experienced grade 3 or 4 immune‐related adverse events (IRAEs), including one case each of pleuropericarditis and myocarditis (both grade 3) [3]. Historically, patients with AD have been omitted from ICI trials due to concerns about flaring their baseline disease, leading to limited data on ICI safety and efficacy in this population. However, the expanding role of ICI in multiple malignancies suggests that an AD diagnosis alone should not preclude their use. A recent meta‐analysis by Lopez‐Olivo et al. [9], which examined 23,897 cancer patients with pre‐existing AD across 95 studies, found that ICI therapy slightly increased the risk of IRAEs (relative risk 1.3, 95% CI 1.0–1.6), although most were mild (grade < 3), manageable with corticosteroids, and did not affect tumour response. In our case, dual ICI therapy did not exacerbate idiopathic pericarditis, further supporting the cautious but feasible application of ICI in individuals with AD under close clinical surveillance.

This case highlights the exceptional nature of MPeM and raises the possibility that long‐standing pericardial inflammation, along with treatment‐related immunosuppression, might play a role in its pathogenesis. Additionally, the use of dual ICI in a patient with pre‐existing autoimmune pericarditis, without exacerbation of her baseline condition, and response of the disease suggests a potential therapeutic strategy worth further exploration.

## Author Contributions

Beatriz Grau Mirete made substantial contributions to the conception, design, and acquisition of data, and was primarily responsible for drafting the initial manuscript. Paula Rodriguez Paya, Mariano Martinez Marin, Asia Ferrández Arias, and Miguel Borregón Rivilla contributed significantly to data collection, analysis, and interpretation, as well as critical revision of the manuscript for important intellectual content. Antonio David Lazaro Sanchez, Pedro Morillas Blasco, Alvaro Rodriguez‐Lescure, and Javier David Benítez Fuentes served as supervisors, providing critical review and approval of the final version to be published, with Javier David Benítez Fuentes acting as the senior author and corresponding author. All authors reviewed and approved the final manuscript before submission, agreeing to be accountable for all aspects of the work.

## Ethics Statement

The authors declare that written informed consent was obtained for the publication of this manuscript and accompanying images using the consent form provided by the journal.

## Conflicts of Interest

Asia Ferrández Arias has received travel, accommodations, or expenses from Pfizer. Alvaro Rodriguez‐Lescure has served in a consulting or advisory role for Roche, Pfizer, Novartis, MSD, AstraZeneca Spain, Daiichi‐Sankyo, Seagen, Pierre Fabre, and Lilly, has participated in speakers' bureaus for Roche, Novartis, Lilly, Pfizer, AstraZeneca Spain, Daiichi‐Sankyo, and Seagen, has received research funding from Roche, Pfizer, Novartis, Lilly, Zymeworks, Bristol Myers Squibb, AstraZeneca Spain, and Radius Health (all to the institution), and has received travel, accommodations, or expenses from Roche and Pfizer. No other potential conflicts of interest were reported.

## Data Availability

The data that support the findings of this study are available on request from the corresponding author. The data are not publicly available due to privacy or ethical restrictions.
